# Psychometric Evaluation of a Fear of COVID-19 Scale in China: Cross-sectional Study

**DOI:** 10.2196/31992

**Published:** 2022-03-02

**Authors:** Edmond P H Choi, Wenjie Duan, Daniel Y T Fong, Kris Y W Lok, Mandy Ho, Janet Y H Wong, Chia-Chin Lin

**Affiliations:** 1 School of Nursing The University of Hong Kong Hong Kong China (Hong Kong); 2 Social and Public Administration School East China University of Science and Technology Shanghai China; 3 School of Nursing College of Nursing Taipei Medical University Taipei Taiwan

**Keywords:** Chinese, COVID-19, fear, psychometric, validation, scale, mental health, information, cross-sectional, validity, reliability, support

## Abstract

**Background:**

At the very beginning of the COVID-19 pandemic, information about fear of COVID-19 was very limited in Chinese populations, and there was no standardized and validated scale to measure the fear associated with the pandemic.

**Objective:**

This cross-sectional study aimed to adapt and validate a fear scale to determine the levels of fear of COVID-19 among the general population in mainland China and Hong Kong.

**Methods:**

A web-based questionnaire platform was developed for data collection; the study instruments were an adapted version of the 8-item Breast Cancer Fear Scale (“Fear Scale”) and the 4-item Patient Health Questionnaire. The internal construct validity, convergent validity, known group validity, and reliability of the adapted Fear Scale were assessed, and descriptive statistics were used to summarize the participants’ fear levels.

**Results:**

A total of 2822 study participants aged 18 years or older were included in the analysis. The reliability of the adapted scale was satisfactory, with a Cronbach α coefficient of .93. The item-total correlations corrected for overlap were >0.4, confirming their internal construct validity. Regarding convergent validity, a small-to-moderate correlation between the Fear Scale and the 4-item Patient Health Questionnaire scores was found. Regarding known group validity, we found that the study participants who were recruited from Hong Kong had a higher level of fear than the study participants from mainland China. Older adults had a higher level of fear compared with younger adults. Furthermore, having hypertension, liver disease, heart disease, cancer, anxiety, and insomnia were associated with a higher fear level. The descriptive analysis found that more than 40% of the study participants reported that the thought of COVID-19 scared them. About one-third of the study participants reported that when they thought about COVID-19, they felt nervous, uneasy, and depressed.

**Conclusions:**

The psychometric properties of the adapted Fear Scale are acceptable to measure the fear of COVID-19 among Chinese people. Our study stresses the need for more psychosocial support and care to help this population cope with their fears during the pandemic.

## Introduction

In December 2019, the novel coronavirus disease 2019 (COVID-19) emerged in Wuhan City, China [1]. The outbreak rapidly evolved into a global pandemic [2], affecting more than 190 countries and regions [3]. The COVID-19 pandemic continues to spread on a global scale. As of December 20, 2021, there have been more than 271 million confirmed cases of COVID-19 worldwide, with more than 5 million deaths [4]. The COVID-19 pandemic has lasted for almost 2 years and is still ongoing. With time, an increasing number of COVID-19 variants was reported globally [5].

COVID-19 is not only life-threatening, but it also leads to psychological distress [6-10]. The concomitant public health measures such as quarantines, social distancing, and lockdowns can also increase psychosocial distress. Since the start of the COVID-19 pandemic, a plethora of research studies have been conducted to examine the psychological status of people during the pandemic. A meta-analysis of 55 peer-reviewed studies found that the prevalence of depression was 16%, the prevalence of anxiety was 15%, the prevalence of insomnia was 24%, and the prevalence of posttraumatic stress disorder was 22% [11]. Another meta-analysis of the prevalence of stress, anxiety, and depression among the general population during the COVID-19 pandemic found that the prevalence of stress was 29.6%, that of anxiety was 32%, and that of depression 34% [12]. Moreover, compared with studies conducted in Europe, those conducted in Asia found a higher prevalence of anxiety (Asia 33% vs Europe 24%) and depression (Asia 35% vs Europe 32%) [12]. These studies suggest that the pandemic substantially jeopardizes the psychological well-being of the general population [11,12].

In addition to anxiety, depression, and stress, fear is also a common psychological response to COVID-19 [13]. In brief, fear is an adaptive emotion that helps defend against potential danger [14]. Fear may occur in response to specific stimuli in the present environment or in anticipation of future or imagined situations that pose a threat to oneself [15]. During the COVID-19 pandemic, people may experience the fear of contracting the infection and a feeling of uncertainty. Fear can be beneficial because it can motivate people to engage in preventive behaviors, such as hand hygiene and mask wearing [16]. However, excessive fear can be maladaptive, leading to psychological distress. For example, fear of COVID-19 may exacerbate preexisting mental health and psychiatric conditions [17]. In extreme situations, fear may lead to suicidal ideation [18]. Excessive fear can cause irrational behaviors, such as panic buying [19].

It is noteworthy that the COVID-19 pandemic has reignited the fear resulting from the 2003 severe acute respiratory syndrome outbreak for many people in mainland China and Hong Kong. This adverse experience was unique to those populations. The fear levels of people in mainland China and Hong Kong may therefore be different from those in populations that did not undergo that adverse experience. Assessing and managing fear is a crucial component of outbreak control and health promotion [20].

In this study, the 8-item Breast Cancer Fear Scale developed by Champion et al [21] was used. We chose this instrument to measure fear levels for several reasons. First, at the very beginning of the pandemic, there was no standardized and validated study instrument specifically developed to measure fear levels related to COVID-19. For example, the Fear of COVID-19 Scale developed by Ahorsu and colleagues [13] was not available when we planned this study. Second, the 8-item Breast Cancer Fear Scale (“Fear Scale”) was one of the few instruments available to measure fear among the Hong Kong Chinese population [22]. Furthermore, even though the Fear Scale was originally developed to measure fear related to breast cancer, the question items are generic and comprehensive. The Fear Scale covers common responses to fear such as feeling scared, nervous, upset, depressed, jittery, uneasy, and anxious, as well as having heart palpitations. According to a study in Canada, many participants felt uneasy, distressed, anxious, and nervous due to the COVID-19 pandemic [23]. A study in Slovakia reported an overall increase in negative feeling such as feeling upset, scared, and afraid during the COVID-19 pandemic [24,25]. The items of the Fear Scale should be applicable and appropriate to measure the fear related to COVID-19.

This study aimed to adapt and validate the Fear Scale to determine the levels of fear of COVID-19 in mainland China and Hong Kong. With the information on how an individual fears COVID-19, health care providers can design appropriate psychosocial interventions to meet the public’s needs.

## Methods

### Study Design, Participants, and Sampling

An international study was conducted, which aimed to examine the global impact of the COVID-19 pandemic on lifestyle behaviors, fear, depression, and perceived needs of communities [26,27]. The study was conducted in 30 countries across the globe. It is a cross-sectional web-based survey design. Moreover, a web-based questionnaire platform was developed for data collection [28].

For this analysis, only data collected in mainland China and Hong Kong between July 2020 and January 2021 were used. Study eligibility criteria included (1) aged ≥18 years; (2) being able to read and understand Chinese; and (3) having an internet access. To recruit more people with diverse sociodemographic backgrounds, multiple recruitment strategies were used to recruit study participants. The study participants were recruited by survey service providers, social media platforms such as Facebook, WeChat, and Twitter, and snowball sampling, in which the existing study participants helped to recruit additional participants to join this study. To encourage more people to complete the survey, for each completed questionnaire, HK $1 (US $0.13) would be donated to the Red Cross in the respondent’s region.

The study protocol has been published elsewhere [26]. The study was approved by the institutional review board of the University of Hong Kong/Hospital Authority Hong Kong West Cluster (reference UW 20-272). All the procedures involving human participants in this study were conducted in accordance with the ethical standards of the institutional review board and the 1964 Declaration of Helsinki and its later amendments. Electronic informed consent was obtained from each study participant.

### Outcomes and Instruments

The primary outcome of the study was the fear of COVID-19. To measure the fear of COVID-19, we adapted the 8-item Breast Cancer Fear Scale developed by Champion et al [21] for this study. The study instrument was originally developed to measure women’s emotional responses to breast cancer. In the scale developed by Champion et al [21], a 5-point Likert scale is used (1=strongly disagree, 2=disagree, 3=neutral, 4=agree, and 5=strongly agree); a higher score indicates a higher level of fear. The total score of the instrument is the sum of each item. In this paper, we changed the words “breast cancer” to “COVID-19” in all of the following 8 items: (1) the thought of COVID-19 scares me; (2) when I think about COVID-19, I feel nervous; (3) when I think about COVID-19, I get upset; (4) when I think about COVID-19, I get depressed; (5) when I think about COVID-19, I get jittery; (6) when I think about COVID-19, my heart beats faster; (7) when I think about COVID-19, I feel uneasy; and (8) when I think about COVID-19, I feel anxious.

The face validity of the adapted instrument was evaluated by an expert panel of this study.

The 4-item Patient Health Questionnaire (PHQ-4), which measures anxiety and depressive symptoms, was administered to evaluate the convergent validity of the Fear Scale. The PHQ-4 includes the 2-item Generalized Anxiety Disorder scale and the 2-item Patient Health Questionnaire (PHQ-2). A 4-point Likert scale is used (0=not at all; 1=several days; 2=more than half the days; and 3=nearly every day). The summary score of the PHQ-4 ranges from 0 to 12, with a higher score indicating greater anxiety and depressive symptoms. The PHQ-4 was validated in Chinese adults [29]. The study supported its 2-factor model and reliability [29]. Cronbach α coefficient was .87 for the PHQ-4, .80 for the 2-item Generalized Anxiety Disorder, and .80 for the PHQ-2 in this study.

A structured questionnaire was used to collect sociodemographic factors such as age, gender, and comorbidities.

### Data Analysis

The internal construct validity, convergent validity, known group validity, and reliability of the Fear Scale were assessed. The internal construct validity was evaluated using the corrected item-total correlation; a correlation coefficient of ≥0.4 indicated adequate internal construct validity. The convergent validity of the Fear Scale was determined by calculating the Pearson correlation coefficient between the total score of the Fear Scale and the total score of the PHQ-4. It was hypothesized that an absolute value Pearson correlation coefficient of at least 0.3 was required [30]. To evaluate the known group validity, independent *t* tests were used to compare the mean score of the Fear Scale between (1) people recruited from mainland China and people recruited from Hong Kong [31]; (2) people aged 18-59 years and people aged 60 years or older [15]; and (3) male and female participants [32]. A study among Chinese university students reported that students in mainland China had lower fear of instability related to the COVID-19 pandemic when compared with students in Hong Kong [31]. Another study among pregnant women and new mothers reported that compared with the study participants in mainland China, the level of fear related to the COVID-19 pandemic was significantly higher among study participants in Hong Kong [33]. A study in Singapore found that older age was associated with greater fear of COVID-19 [15]. Another study in the Spanish population found that fear was higher among women than among men [32]. Besides, a study in Turkey reported that the COVID-19 fear scores were higher among people with a chronic disease [34]. Therefore, we also compared the mean score of the Fear Scale between people with and without the following chronic diseases, which were highly prevalent in Chinese populations: (1) hypertension; (2) diabetes; (3) liver disease; (4) heart disease; (5) stroke; (6) chronic obstructive pulmonary disease; (7) cancer; (8) depression; (9) anxiety; and (10) insomnia. Cohen *d* effect sizes were also calculated. The interpretation of the effect sizes was as follows: trivial (<0.2), small (≥0.2 to <0.5), moderate (≥0.5 to <0.8) and large (≥0.8). Finally, descriptive statistics were used to describe the fear levels of the study participants. Furthermore, multiple linear regression analysis was used to explore the known associations between sociodemographic and clinical factors, on the one hand, and the Fear Scale, on the other.

## Results

### Participants’ Characteristics

A total of 2822 study participants aged 18 years or older were included in the analysis, of whom 61% (n=1721) were female and 38.8% (n=1096) were male. Three-quarters, 75.6% (n=2133), were recruited from Hong Kong while 24.4% (n=689) were recruited from mainland China. Almost half (1450, 51.4%) of the participants were married. Hypertension (294, 10.4%) was the most prevalent reported chronic disease, followed by diabetes (155, 5.5%), insomnia (90, 3.2%) and heart disease (62, 2.2%). Table 1 shows the participants’ characteristics of the study.

**Table 1:** Sociodemographic and clinical profile (N=2822).

Characteristics		Values, n (%)
**Geographical areas**		
	China	689 (24.4)
	Hong Kong	2133 (75.6)
**Age groups (years)**		
	18-59	2547 (90.3)
	≥60	275 (9.7)
**Genders**		
	Female	1721 (61)
	Male	1096 (38.8)
	Nonbinary	5 (0.2)
**Marital status**		
	Single	1265 (44.8)
	Married or cohabitation or common-law	1450 (51.4)
	Separated or divorced or widowed	107 (3.8)
**Educational level**		
	Primary or below	169 (6)
	Secondary	1191 (42.2)
	College	289 (10.2)
	Associate degree	204 (7.2)
	Bachelor’s degree	780 (27.6)
	Master’s degree or above	189 (6.7)
**Employment status**		
	Nonworking	589 (20.9)
	Working	1682 (59.6)
	Student	551 (19.5)
**Chronic diseases**		
	Hypertension	294 (10.4)
	Diabetes	155 (5.5)
	Liver disease	50 (1.8)
	Heart disease	62 (2.2)
	Stroke	18 (0.6)
	Chronic obstructive pulmonary disease	36 (1.3)
	Cancer	16 (0.6)
	Insomnia	90 (3.2)
	Depression	43 (1.5)
	Anxiety	44 (1.6)

### Reliability and Validity of the Fear Scale

The mean score of the Fear Scale was 23.60 (SD 6.64), and the Cronbach α coefficient was .93. The corrected item-total correlations were >0.7 for all items. Table 2 shows the results of the internal consistency and internal construct validity. The Pearson correlation coefficient between the Fear Scale and PHQ-4 scores was 0.23 (*P*<.001). Table 3 shows the results of the convergent validity.

**Table 2:** Descriptive statistics, internal construct validity, and reliability of the Fear Scale (N=2822).

	Corrected item-total correlation (n=2821)	Mean (SD)^a^	Strongly disagree, n (%)	Disagree, n (%)	Neutral, n (%)	Agree, n (%)	Strongly agree, n (%)	Missing, n (%)
The thought of COVID-19 scares me	0.71	3.27 (0.98)	151 (5.4)	451 (16)	890 (31.5)	1145 (40.6)	185 (6.6)	N/A^b^
When I think about COVID-19, I feel nervous	0.73	3.11 (0.99)	187 (6.6)	527 (18.7)	1076 (38.1)	862 (30.6)	170 (6)	N/A
When I think about COVID-19, I get upset	0.75	2.99 (1.02)	225 (8)	642 (22.8)	1083 (38.4)	694 (24.6)	178 (6.3)	N/A
When I think about COVID-19, I get depressed	0.80	2.99 (1.03)	235 (8.3)	656 (23.3)	1007 (35.7)	760 (26.9)	163 (5.8)	1 (0.04)
When I think about COVID-19, I get jittery	0.71	2.63 (1.04)	449 (15.9)	810 (28.7)	992 (35.2)	480 (17)	91 (3.2)	N/A
When I think about COVID-19, my heart beats faster	0.70	2.64 (1.04)	426 (15.1)	823 (29.2)	1006 (35.6)	463 (16.4)	104 (3.7)	N/A
When I think about COVID-19, I feel uneasy	0.78	3.03 (1.04)	256 (9.1)	560 (19.8)	1003 (35.5)	854 (30.3)	149 (5.3)	N/A
When I think about COVID-19, I feel anxious	0.80	2.95 (1.03)	279 (9.9)	585 (20.7)	1101 (39)	719 (25.5)	138 (4.9)	N/A
Cronbach alpha	0.93	N/A	N/A	N/A	N/A	N/A	N/A	N/A

^a^A higher score means a higher level of fear.

^b^N/A: not applicable.

**Table 3:** Convergent validity of the Fear Scale (N=2822).

Scale	Population, n	Mean (SD)^a^	Pearson correlation coefficient	*P* value
PHQ-4^b^ anxiety subscale or GAD-2^c^	2822	1.22 (1.26)	—^d^	—
PHQ-4 depression subscale or PHQ-2^e^	2821	1.10 (1.30)	—	—
PHQ-4 total score	2821	2.32 (2.37)	—	—
Fear Scale total score	2821	23.60 (6.64)	—	—
PHQ-4 anxiety subscale or GAD-2	2821	—	0.25	<.001
PHQ-4 depression subscale PHQ-2	2820	—	0.19	<.001
PHQ-4 total score	2820	—	0.23	<.001

^a^A higher score means a higher level of fear or anxiety or depression.

^b^PHQ-4: 4-item Patient Health Questionnaire.

^c^GAD-2: two-item Generalized Anxiety Disorder scale.

^d^Not available.

^e^PHQ-2: 2-item Patient Health Questionnaire.

With respect to the known group comparisons, the results of the independent *t* tests showed that the study participants who were recruited from Hong Kong had a higher level of fear compared with the study participants from mainland China (Cohen *d* effect size 0.24). Furthermore, older adults (60 years or above) had a higher level of fear than younger adults (Cohen *d* effect size 0.39). Study participants with cancer (Cohen *d* effect size 0.58), heart disease (Cohen *d* effect size 0.44), hypertension (Cohen *d* effect size 0.36), liver disease (Cohen *d* effect size 0.33), insomnia (Cohen *d* effect size 0.33), and anxiety (Cohen *d* effect size 0.28) had a higher level of fear than those without such conditions. Table 4 and Table 5 show the results of the known group comparisons by independent *t* test.

The results of multiple linear regression are shown in the Multimedia Appendix 1.

**Table 4:** Known group comparison: sociodemographic factors (N=2822).

Sociodemographic factors		n (%)		Fear Scale, mean (SD)		*P* value	Cohen *d* effect size
**Location**						<.001	0.24
	Mainland China	688 (24.4)	22.40 (6.49)				
	Hong Kong	2133 (75.6)	23.98 (6.64)				
**Gender**						.80	0.01
	Female	1720 (60.9)	23.58 (6.60)				
	Male	1096 (38.8)	23.64 (6.69)				
**Age (years)**						<.001	0.39
	18-59	2546 (90.2)	23.34 (6.56)				
	≥60	275 (9.7)	25.98 (6.86)				

**Table 5:** Known group comparison: clinical factors (N=2822).

Clinical factors		n (%)		Fear Scale, mean (SD)		*P* value		Cohen *d* effect size
**Hypertension**						<.001		0.36
	No	2527 (89.5)	23.35 (6.59)					
	Yes	294 (10.4)	25.73 (6.68)					
**Diabetes**						.11		0.13
	No	2666 (94.5)	23.55 (6.63)					
	Yes	155 (5.5)	24.43 (6.77)					
**Liver disease**						.02		0.33
	No	2771 (98.2)	23.56 (6.63)					
	Yes	50 (1.8)	25.78 (6.96)					
**Heart disease**						<.001		0.44
	No	2759 (97.8)	23.53 (6.61)					
	Yes	62 (2.2)	26.61 (7.27)					
**Stroke**						.16		0.31
	No	2803 (99.3)	23.58 (6.63)					
	Yes	18 (0.6)	25.78 (7.40)					
**Chronic obstructive pulmonary disease**						.28		0.19
	No	2785 (98.7)	23.58 (6.65)					
	Yes	36 (1.3)	24.78 (5.83)					
**Cancer**						.04		0.58
	No	2805 (99.4)	23.58 (6.64)					
	Yes	16 (0.6)	26.94 (4.74)					
**Depression**						.17		0.20
	No	2778 (98.4)	23.58 (6.63)					
	Yes	43 (1.5)	24.98 (7.40)					
**Anxiety**						.04		0.28
	No	2777 (98.4)	23.57 (6.61)					
	Yes	44 (1.6)	25.61 (8.01)					
**Insomnia**						.002		0.33
	No	2731 (96.8)	23.53 (6.63)					
	Yes	90 (3.2)	25.70 (6.47)					

### The Fear Levels of the Study Participants

In total, 47.1% (n=1330) of the participants reported that the thought of COVID-19 scared them. Moreover, 36.6% (n=1032) of the study participants reported that they felt nervous when they thought about COVID-19. About one-third of the participants reported that they felt uneasy (1003, 35.5%) and became depressed (923, 32.7%) when they thought about COVID-19. The descriptive statistics of the Fear Scale are shown in Table 2.

We also separated the analysis between data collected in Hong Kong and those collected in China. Those results are shown in Multimedia Appendix 1.

## Discussion

### Principal Results

In the first part of this study, we assessed the psychometric properties in terms of internal construct validity, convergent validity, known group validity, and reliability of the Fear Scale. The Cronbach α coefficient was .93, which is far larger than the recommended cut-off value of .7. This finding supports the general agreement between the 8 items that make up the composite score of the scale to measure the fear related to COVID-19. Moreover, we found that all the coefficients of the item-total correlation, corrected for overlaps, were larger than 0.4, supporting the internal construct validity of the modified scale. These results supported the suggestion that all individual items measured the same construct as that measured by the other items. With regards to the study’s convergent validity, we found a small-to-moderate correlation between the total score of the Fear Scale and the total score of the PHQ-4. Another important finding of this study was that participants with a chronic disease had a higher fear level than those without a chronic disease. Particularly, we found that hypertension, liver disease, heart disease, cancer, anxiety, and insomnia were associated with a higher fear level.

### Limitations

There were some limitations in this study. First, the study was conducted in mainland China and Hong Kong. Therefore, the study findings may not be transferable to other geographic areas in which the severity of COVID-19, case fatality rate, and infection control measures are different. We expect that the fear level would be even higher in areas where the severity and case fatality rate of COVID-19 were more severe. Second, we could not explore the trajectory of the fear levels over time due to the cross-sectional nature of the study. Third, we adapted the Breast Cancer Fear Scale in this study; thus, some of the constructs related to COVID-19 could not be measured. However, as previously mentioned, there was no validated fear scale specific to COVID-19 when we planned our study. Fourth, regarding the reliability of the scale, we only evaluated its internal consistency. We were not able to evaluate the test-retest reliability of the scale due to the cross-sectional design of the study. Fifth, regarding the known group comparison, the sample size of some subgroups was small such as that of patients with diabetes and depression. There might be insufficient statistical power to detect the differences between groups. Finally, we used a web-based questionnaire platform to collect the data. People with low computer literacy would probably be excluded from the study. Accordingly, the potential sampling bias should be noted.

### Comparisons With Prior Work

We found that the total score of the Fear Scale had a higher correlation with the PHQ-4 anxiety subscale than with the PHQ-4 depression subscale. In fact, there are distinct differences in psychological features between fear and depression. According to Witte [35], fear is conceptualized as negatively toned emotion accompanied by a high level of physiologic arousal stimulated by a threat. Fear can be expressed as a physiological arousal, such as feeling “jittery” and “heart beating faster,” through verbal self-reports of fear (eg, “I feel scared”) and overt acts that exhibit fear, such as facial expression [21]. These emotional and physiological reactions to perceived threats are fundamentally different from those of depression, which is manifested through the following 4 symptom clusters: (1) emotional symptoms such as feeling sad and worthless; (2) cognitive symptoms such as a negative view of the self and hopelessness; (3) motivational symptoms such as a lack of incentive; and (4) somatic symptoms such as a loss of appetite and sleep disturbances [36,37]. Additionally, it was suggested that fear and anxiety are largely distinct emotions. A meta-analysis reported only a moderate (*r*=0.32) relationship between measures of trait fear and anxiety [38]. Fear is an aversive psychological state during which an individual is motivated to escape a specific and imminent threat. The characteristics of fear include short-lived arousal that quickly dissipates after the threat is avoided. By contrast, anxiety is an aversive psychological state that occurs while an individual approaches an ambiguous and uncertain threat. Hypervigilance and hyperarousal are the typical behaviors characteristic of anxiety [38]. The small-to-moderate correlation between the Fear Scale and the PHQ-4 further supported the need for this study, which adapted and validated the Fear Scale to measure the fear of COVID-19. Besides, compared with study participants recruited in Hong Kong, those recruited from mainland China had a higher PHQ-4 score but lower Fear Scale total score. This finding further suggested that the constructs of fear and anxiety are different.

Participants with a chronic disease had a higher fear level than those without one. This finding was consistent with that of a matched case-control study, which found that the prevalence of anxiety symptoms and depressive symptoms and the level of stress were significantly higher among those with preexisting chronic health conditions (59%, 71.6%, and 73.7%, respectively) compared with controls (25.6%, 31.1%, and 43.3%, respectively) [39]. Evidence has suggested that the presence of comorbid chronic conditions would increase the risk of death from COVID-19 [40-42]. Moreover, one major concern with the COVID-19 pandemic was its impacts on the routine use of health care services especially for individuals with comorbidities [43]. Service disruptions due to cancellations of elective care and lockdowns hindering access to health care facilities, in addition to the diffidence of patients with a chronic disease in seeking assistance for fear of risking iatrogenic exposure, altogether increased the psychological burden of patients with a chronic disease. Thus, it was not surprising that people with a chronic disease had a higher fear level than those without.

In this study, more than 40% of the study participants reported that the thought of COVID-19 scared them. About one-third of the study participants reported that when they thought about COVID-19, they felt nervous, uneasy, and depressed. No doubt, the COVID-19 pandemic was very stressful for people and the communities in general [7]; the fear of infection was very common during the pandemic. Furthermore, people were worried that the health care system could not cope with the COVID-19 pandemic, that there were not enough hospital beds and ventilators to handle the rising number of COVID-19 cases. Another concern weighing on people’s minds was the COVID-19 recession. Fear of the COVID-19 pandemic can be overwhelming and cause strong emotions [7]. It was also noteworthy that the COVID-19 pandemic rekindled fears of the 2003 severe acute respiratory syndrome epidemic in mainland China and Hong Kong.

### Implications

First, based on the psychometric evaluation, we found that the adapted scale was a valid and reliable measure to assess the level of fear related to COVID-19. Further studies can use this scale to longitudinally monitor the fear level in different communities. Second, given the high fear levels found in the study sample, it is required to provide psychosocial care for the general public to diminish the psychological burden of the pandemic. Third, the findings call for the need to provide more psychosocial care for chronic disease patients and older adults.

### Conclusion

This study found that the psychometric properties of the Fear Scale were acceptable to evaluate the fear level of the general Chinese population. Our descriptive analysis found that more than 40% of the study participants reported nervousness when they thought about COVID-19. About one-third of the study participants reported that when they thought about COVID-19, they felt nervous, uneasy, and depressed. Additionally, we found that people with a chronic disease reported a higher fear level than those without. The findings call for the need to provide more psychosocial care for chronic disease patients and older adults.

## Appendix

Multimedia Appendix 1 Supplementary tables.

## Abbreviations

PHQ: Patient Health Questionnaire

## References

[1] Li Q, Guan X, Wu P, Wang X, Zhou L, Tong Y, Ren R, Leung KS, Lau EH, Wong JY, Xing X, Xiang N, Wu Y, Li C, Chen Q, Li D, Liu T, Zhao J, Liu M, Tu W, Chen C, Jin L, Yang R, Wang Q, Zhou S, Wang R, Liu H, Luo Y, Liu Y, Shao G, Li H, Tao Z, Yang Y, Deng Z, Liu B, Ma Z, Zhang Y, Shi G, Lam TT, Wu JT, Gao GF, Cowling BJ, Yang B, Leung GM, Feng Z (2020). Early Transmission Dynamics in Wuhan, China, of Novel Coronavirus-Infected Pneumonia. N Engl J Med.

[2] Cucinotta D, Vanelli M (2020). WHO Declares COVID-19 a Pandemic. Acta Biomed.

[3] COVID-19 Dashboard. John Hopkins University.

[4] WHO Coronavirus Disease (COVID-19) Dashboard. World Health Organization.

[5] Lam HY, Lau CCA, Wong CH, Lee KYK, Yip SL, Tsang KLA, Cheng KCP, Au KWA, Ng HLK, Chuang SK, Lam MKR (2022). A review of epidemiology and public health control measures of COVID-19 variants in Hong Kong, December 2020 to June 2021. IJID Regions.

[6] Qiu J, Shen B, Zhao M, Wang Z, Xie B, Xu Y (2020). A nationwide survey of psychological distress among Chinese people in the COVID-19 epidemic: implications and policy recommendations. Gen Psychiatr.

[7] Choi EPH, Hui BPH, Wan EYF (2020). Depression and Anxiety in Hong Kong during COVID-19. Int J Environ Res Public Health.

[8] Choi EPH, Hui BPH, Wan EYF, Kwok JYY, Tam THL, Wu C (2021). COVID-19 and Health-Related Quality of Life: A Community-Based Online Survey in Hong Kong. Int J Environ Res Public Health.

[9] Allen R, Prescott J, McHugh S, Carson J (2021). Loneliness and mental health at the early stages of the Covid-19 pandemic in England. Health Soc Care Community.

[10] Maleku A, Kim YK, Kirsch J, Um MY, Haran H, Yu M, Moon SS (2021). The hidden minority: Discrimination and mental health among international students in the US during the COVID-19 pandemic. Health Soc Care Community.

[11] Cénat Jude Mary, Blais-Rochette C, Kokou-Kpolou CK, Noorishad P, Mukunzi JN, McIntee S, Dalexis RD, Goulet M, Labelle PR (2021). Prevalence of symptoms of depression, anxiety, insomnia, posttraumatic stress disorder, and psychological distress among populations affected by the COVID-19 pandemic: A systematic review and meta-analysis. Psychiatry Res.

[12] Salari N, Hosseinian-Far A, Jalali R, Vaisi-Raygani A, Rasoulpoor S, Mohammadi M, Rasoulpoor S, Khaledi-Paveh B (2020). Prevalence of stress, anxiety, depression among the general population during the COVID-19 pandemic: a systematic review and meta-analysis. Global Health.

[13] Ahorsu DK, Lin C, Imani V, Saffari M, Griffiths MD, Pakpour AH (2020). The Fear of COVID-19 Scale: Development and Initial Validation. Int J Ment Health Addict.

[14] Dunsmoor JE, Paz R (2015). Fear Generalization and Anxiety: Behavioral and Neural Mechanisms. Biol Psychiatry.

[15] Han MFY, Mahendran R, Yu J (2021). Associations Between Fear of COVID-19, Affective Symptoms and Risk Perception Among Community-Dwelling Older Adults During a COVID-19 Lockdown. Front Psychol.

[16] Harper C, Satchell L, Fido D, Latzman R (2021). Functional Fear Predicts Public Health Compliance in the COVID-19 Pandemic. Int J Ment Health Addict.

[17] Ho CS, Chee CY, Ho RC (2020). Mental Health Strategies to Combat the Psychological Impact of Coronavirus Disease 2019 (COVID-19) Beyond Paranoia and Panic. Ann Acad Med Singap.

[18] Dsouza DD, Quadros S, Hyderabadwala ZJ, Mamun MA (2020). Aggregated COVID-19 suicide incidences in India: Fear of COVID-19 infection is the prominent causative factor. Psychiatry Res.

[19] Arafat SY, Kar SK, Marthoenis M, Sharma P, Hoque Apu E, Kabir R (2020). Psychological underpinning of panic buying during pandemic (COVID-19). Psychiatry Res.

[20] Sit S, Lam T, Lai A, Wong B, Wang M, Ho S (2021). Fear of COVID-19 and its associations with perceived personal and family benefits and harms in Hong Kong. Transl Behav Med.

[21] Champion VL, Skinner CS, Menon U, Rawl S, Giesler RB, Monahan P, Daggy J (2004). A breast cancer fear scale: psychometric development. J Health Psychol.

[22] Leung DY, Wong EM, Chan CW (2014). Adapting Champion's Breast Cancer Fear Scale to colorectal cancer: psychometric testing in a sample of older Chinese adults. Eur J Oncol Nurs.

[23] Ferguson KN, Coen SE, Tobin D, Martin G, Seabrook JA, Gilliland JA (2021). The mental well-being and coping strategies of Canadian adolescents during the COVID-19 pandemic: a qualitative, cross-sectional study. CMAJ Open.

[24] Šrol Jakub, Ballová Mikušková Eva, Čavojová Vladimíra (2021). When we are worried, what are we thinking? Anxiety, lack of control, and conspiracy beliefs amidst the COVID-19 pandemic. Appl Cogn Psychol.

[25] Ballová Mikušková E, Verešová M (2020). Distance education during COVID-19: The perspective of Slovak teachers. PEC.

[26] Lok KY, Fong DYT, Wong JY, Ho M, Choi EP, Pandian V, Davidson PM, Duan W, Tarrant M, Lee JJ, Lin C, CARE group (2021). International survey for assessing COVID-19's impact on fear and health: study protocol. BMJ Open.

[27] Deek H, El Nayal M, Alabdulwahhab K, Ahmad M, Shaik R, Alzahrani M, Elmahdi I, Youssef N, Alboraie M, Fong DY, Choi EPH, Chan BKY, Omar N (2021). A multi-centric study on validation of the Fear Scale for COVID-19 in five Arabic speaking countries. Brain Behav.

[28] CARE: An international survey for assessing COVID-19’s impact on fear and health. School of Nursing, The University of Hong Kong.

[29] Guo N, Luk TT, Ho SY, Lee JJ, Shen C, Oliffe J, Chan SS, Lam TH, Wang MP (2020). Problematic Smartphone Use and Mental Health in Chinese Adults: A Population-Based Study. Int J Environ Res Public Health.

[30] Dunlow N, Phillips C, Broder HL (2007). Concurrent validity of the COHIP. Community Dent Oral Epidemiol.

[31] Feng S, Zhang Q, Ho SMY (2021). Fear and anxiety about COVID-19 among local and overseas Chinese university students. Health Soc Care Community.

[32] Sánchez-Teruel David, Robles-Bello MA, Lara-Cabrera M, Valencia-Naranjo N (2022). Gender implications of the Fear of COVID-19 Scale in the Spanish population: A validation study. Psychol Trauma.

[33] Fan HSL, Choi EPH, Ko RWT, Kwok JYY, Wong JYH, Fong DYT, Shek NWM, Ngan HYS, Li J, Huang Y, Ouyang Y, Lok KYW (2021). COVID-19 related fear and depression of pregnant women and new mothers. Public Health Nurs.

[34] Özmen Sümeyye, Özkan Okan, Özer Özlem, Yanardağ MZ (2021). Investigation of COVID-19 Fear, Well-Being and Life Satisfaction in Turkish Society. Soc Work Public Health.

[35] Witte K (1992). Putting the fear back into fear appeals: The extended parallel process model. Communication Monographs.

[36] Rosenhan DL, Seligman MEP (1995). Abnormal psychology.

[37] Gullestad SE (2013). One depression or many?. The Scandinavian Psychoanalytic Review.

[38] Sylvers P, Lilienfeld SO, LaPrairie JL (2011). Differences between trait fear and trait anxiety: implications for psychopathology. Clin Psychol Rev.

[39] Sayeed A, Kundu S, Al Banna Md Hasan, Christopher E, Hasan M, Begum M, Chowdhury Sukanta, Khan Md Shafiqul Islam (2020). Mental Health Outcomes of Adults with Comorbidity and Chronic Diseases during the COVID-19 Pandemic: A Matched Case-Control Study. Psychiatr Danub.

[40] Targher G, Mantovani A, Wang X, Yan H, Sun Q, Pan K, Byrne C, Zheng K, Chen Y, Eslam M, George J, Zheng M (2020). Patients with diabetes are at higher risk for severe illness from COVID-19. Diabetes Metab.

[41] Onder G, Rezza G, Brusaferro S (2020). Case-Fatality Rate and Characteristics of Patients Dying in Relation to COVID-19 in Italy. JAMA.

[42] Yang J, Zheng Y, Gou X, Pu K, Chen Z, Guo Q, Ji R, Wang H, Wang Y, Zhou Y (2020). Prevalence of comorbidities and its effects in patients infected with SARS-CoV-2: a systematic review and meta-analysis. Int J Infect Dis.

[43] Wang J, Spencer A, Hulme C, Corbett A, Khan Z, Vasconcelos Da Silva M, O'Dwyer Siobhan, Wright N, Testad I, Ballard C, Creese B, Smith R (2021). Healthcare utilisation and physical activities for older adults with comorbidities in the UK during COVID-19. Health Soc Care Community.

