# Supplementary material for: Psychometric Evaluation of a Fear of COVID-19 Scale in China: Cross-sectional Study
Source: JMIR Form Res. 2022 Mar 2;6(3):e31992. doi: 10.2196/31992 (PMC8893716; doi:10.2196/31992)
Supplement: Multimedia Appendix 1 [file formative_v6i3e31992_app1.docx]

| **Supplementary Table 1: Descriptive statistics, internal construct validity and reliability of the Fear Scale** | | | | | | | | |
| --- | --- | --- | --- | --- | --- | --- | --- | --- |
| **(Hong Kong, N=2133)** | **Corrected Item-Total Correlation** | **Mean (SD) ^** | **Strongly Disagree** | **Disagree** | **Neutral** | **Agree** | **Strongly Agree** |  |
| The thought of COVID-19 scares me | 0.71 | 3.33 (0.98) | 107 (5.0%) | 325 (15.2%) | 590 (27.7%) | 975 (45.7%) | 136 (6.4%) |  |
| When I think about COVID-19, I feel nervous | 0.72 | 3.11 (1.00) | 145 (6.8%) | 399 (18.7%) | 789 (37.0%) | 675 (31.7%) | 125 (5.9%) |  |
| When I think about COVID-19, I get upset | 0.76 | 2.99 (1.04) | 180 (8.4%) | 481 (22.6%) | 786 (36.6%) | 548 (25.7%) | 138 (6.5%) |  |
| When I think about COVID-19, I get depressed | 0.79 | 3.04 (1.06) | 187 (8.8%) | 454 (21.3%) | 715 (33.5%) | 645 (30.2%) | 132 (6.2%) |  |
| When I think about COVID-19, I get jittery | 0.70 | 2.69 (1.06) | 323 (15.1%) | 577 (27.1%) | 730 (34.2%) | 434 (20.4%) | 69 (3.2%) |  |
| When I think about COVID-19, my heart beats faster | 0.68 | 2.70 (1.06) | 312 (14.6%) | 585 (27.4%) | 747 (35.0%) | 407 (19.1%) | 82 (3.8%) |  |
| When I think about COVID-19, I feel uneasy | 0.77 | 3.10 (1.03) | 184 (8.6%) | 374 (17.5%) | 738 (34.6%) | 721 (33.8%) | 116 (5.4%) |  |
| When I think about COVID-19, I feel anxious | 0.79 | 3.02 (1.02) | 198 (9.3%) | 396 (18.6%) | 815 (38.2%) | 618 (28.9%) | 106 (5.0%) |  |
| Cronbach's Alpha (n=2133) | 0.92 |  |  |  |  |  |  |  |
| **(Mainland China , N=689)** |  |  |  |  |  |  |  |  |
| The thought of COVID-19 scares me | 0.70 | 3.08 (0.98) | 44 (6.4%) | 126 (18.3%) | 300 (43.5%) | 170 (24.8%) | 49 (7.1%) |  |
| When I think about COVID-19, I feel nervous | 0.77 | 3.09 (0.98) | 42 (6.1%) | 128 (18.6%) | 287 (41.7%) | 187 (27.1%) | 45 (6.5%) |  |
| When I think about COVID-19, I get upset | 0.75 | 2.96 (0.97) | 45 (6.5%) | 161 (23.4%) | 297 (43.1%) | 146 (21.2%) | 40 (5.8%) |  |
| When I think about COVID-19, I get depressed | 0.80 | 2.82 (0.94) | 48 (7.0%) | 202 (29.3%) | 292 (42.4%) | 115 (16.7%) | 31 (4.5%) | 1 (0.2%) |
| When I think about COVID-19, I get jittery | 0.77 | 2.43 (0.97) | 126 (18.3%) | 233 (33.8%) | 262 (38.0%) | 46 (6.7%) | 22 (3.2%) |  |
| When I think about COVID-19, my heart beats faster | 0.77 | 2.47 (0.97) | 114 (16.6%) | 238 (34.5%) | 259 (37.6%) | 56 (8.1%) | 22 (3.2%) |  |
| When I think about COVID-19, I feel uneasy | 0.79 | 2.81 (1.02) | 72 (10.5%) | 186 (27.0%) | 265 (38.5%) | 133 (19.3%) | 33 (4.8%) |  |
| When I think about COVID-19, I feel anxious | 0.83 | 2.73 (1.00) | 81 (11.8%) | 189 (27.4%) | 286 (41.5%) | 101 (14.7%) | 32 (4.6%) |  |
| Cronbach's Alpha (n=688) | 0.70 | 3.08 (0.98) | 44 (6.4%) | 126 (18.3%) | 300 (43.5%) | 170 (24.8%) | 49 (7.1%) |  |
| Note:  ^A higher score means a higher level of fear  Abbreviation:  SD: Standard deviation | | | | | | | | |

| **Supplementary Table 2: Convergent validity of the Fear Scale** | | | |
| --- | --- | --- | --- |
|  |  | **Hong Kong** | **Mainland China** |
|  |  | **Mean (SD) ^** | **Mean (SD) ^** |
| The PHQ-4 anxiety subscale/ GAD-2 |  | 1.10 (1.17) (n=2133) | 1.57 (1.44) (n=689) |
| The PHQ-4 depression subscale/ PHQ-2 |  | 0.97 (1.21) (n=2133) | 1.51 (1.46) (n=688) |
| The PHQ-4 total score |  | 2.07 (2.20) (n=2133) | 3.09 (2.71) (n=688) |
| The Fear Scale total score |  | 23.98 (6.64) (n=2133) | 22.40 (6.49) (n=688) |
|  |  |  |  |
|  | **The PHQ-4 anxiety subscale/ GAD-2** | **The PHQ-4 depression subscale/ PHQ-2** | **The PHQ-4 total score** |
| Pearson’s correlation coefficient |  |  |  |
| The Fear Scale total score (Hong Kong) | 0.25** (n=2133) | 0.18** (n=2133) | 0.23** (n=2133) |
| The Fear Scale total score (Mainland China) | 0.33** (n=688) | 0.29** (n=687) | 0.33** (n=687) |
| Note:  ^A higher score means a higher level of fear/ anxiety/ depression  ** *P*-value <.001  Abbreviations:  GAD-2: two-item Generalized Anxiety Disorder scale; PHQ-2: two-item Patient Health Questionnaire; PHQ-4: four-item Patient Health Questionnaire; SD: Standard deviation; | | | |

| **Supplementary Table 3: Known-group comparison by independent t-test** | | | | | | | | | |
| --- | --- | --- | --- | --- | --- | --- | --- | --- | --- |
| **Hong Kong** | | | | |  | **Mainland China** | | | |
|  | **Young adults**  **(18 years to 59 years)** | **Older adults**  **60 years of above** |  |  |  |  |  |  |  |
|  | **n=1858** | **n=275** |  |  |  |  |  |  |  |
|  | **Mean (SD)** | **Mean (SD)** | ***P*-value** | **Cohen d effect Size** |  |  |  |  |  |
| **Fear Scale** | 23.69 (6.56) | 25.98 (6.86) | .01 | 0.34 |  |  |  |  |  |
|  | **Female** | **Male** |  |  |  | **Female** | **Male** |  |  |
|  | **n=1189** | **n=943** |  |  |  | **n=531** | **n=153** |  |  |
|  | **Mean (SD)** | **Mean (SD)** | ***P*-value** | **Cohen d effect Size** |  | **Mean (SD)** | **Mean (SD)** | ***P*-value** | **Cohen d effect Size** |
| **Fear Scale** | 24.02 (6.70) | 23.93 (6.56) | .76 | 0.01 |  | 22.58 (6.26) | 21.84 (7.20) | .25 | 0.11 |
|  | **Hypertension** | | | |  | **Hypertension** | | | |
|  | **No** | **Yes** |  |  |  | **No** | **Yes** |  |  |
|  | **n=1852** | **n=281** |  |  |  | **n=675** | **n=13** |  |  |
|  | **Mean (SD)** | **Mean (SD)** | ***P*-value** | **Cohen d effect Size** |  | **Mean (SD)** | **Mean (SD)** | ***P*-value** | **Cohen d effect Size** |
| **Fear Scale** | 23.69 (6.60) | 25.91 (6.64) | .01 | 0.34 |  | 22.41 (6.49) | 21.92 (6.49) | .79 | 0.98 |
|  | **Diabetes** | | | |  | **Diabetes** | | | |
|  | **No** | **Yes** |  |  |  | **No** | **Yes** |  |  |
|  | **n=1982** | **n=151** |  |  |  | **n=684** | **n=4** |  |  |
|  | **Mean (SD)** | **Mean (SD)** | ***P*-value** | **Cohen d effect Size** |  | **Mean (SD)** | **Mean (SD)** | ***P*-value** | **Cohen d effect Size** |
| **Fear Scale** | 23.95 (6.63) | 24.48 (6.83) | .34 | 0.08 |  | 22.40 (6.50) | 22.25 (4.27) | .96 | 0.03 |
|  | **Liver diseases** | | | |  | **Liver diseases** | | | |
|  | **No** | **Yes** |  |  |  | **No** | **Yes** |  |  |
|  | **n=2111** | **n=22** |  |  |  | **n=660** | **n=28** |  |  |
|  | **Mean (SD)** | **Mean (SD)** | ***P*-value** | **Cohen d effect Size** |  | **Mean (SD)** | **Mean (SD)** | ***P*-value** | **Cohen d effect Size** |
| **Fear Scale** | 23.95 (6.63) | 27.77 (6.77) | .01 | 0.57 |  | 22.32 (6.47) | 24.21 (6.81) | .13 | 0.28 |
|  | **Heart diseases** | | | |  | **Heart diseases** | | | |
|  | **No** | **Yes** |  |  |  | **No** | **Yes** |  |  |
|  | **n=2075** | **n=58** |  |  |  | **n=684** | **n=4** |  |  |
|  | **Mean (SD)** | **Mean (SD)** | ***P*-value** | **Cohen d effect Size** |  | **Mean (SD)** | **Mean (SD)** | ***P*-value** | **Cohen d effect Size** |
| **Fear Scale** | 23.91 (6.62) | 26.57 (7.10) | .01 | 0.39 |  | 22.37 (6.46) | 27.25 (10.69) | .13 | 0.55 |
|  | **Stroke** | | | |  | **Stroke** | | | |
|  | **No** | **Yes** |  |  |  |  |  |  |  |
|  | **n=2116** | **n=17** |  |  |  |  |  |  |  |
|  | **Mean (SD)** | **Mean (SD)** | ***P*-value** | **Cohen d effect Size** |  |  |  |  |  |
| **Fear Scale** | 23.97 (6.63) | 25.82 (7.63) | .25 | 0.26 |  |  |  |  |  |
|  | **Chronic obstructive pulmonary disease** | | | |  | **Chronic obstructive pulmonary disease** | | | |
|  | **No** | **Yes** |  |  |  | **No** | **Yes** |  |  |
|  | **n=2105** | **n=28** |  |  |  | **n=680** | **n=8** |  |  |
|  | **Mean (SD)** | **Mean (SD)** | ***P*-value** | **Cohen d effect Size** |  | **Mean (SD)** | **Mean (SD)** | ***P*-value** | **Cohen d effect Size** |
| **Fear Scale** | 23.97 (6.65) | 25.36 (5.94) | .27 | 0.22 |  | 22.39 (6.50) | 22.75 (5.28) | .88 | 0.06 |
|  | **Cancer** | | | |  | **Cancer** | | | |
|  | **No** | **Yes** |  |  |  | **No** | **Yes** |  |  |
|  | **n=2119** | **n=14** |  |  |  | **n=686** | **n=2** |  |  |
|  | **Mean (SD)** | **Mean (SD)** | ***P*-value** | **Cohen d effect Size** |  | **Mean (SD)** | **Mean (SD)** | ***P*-value** | **Cohen d effect Size** |
| **Fear Scale** | 23.97 (6.65) | 26.79 (5.01) | .11 | 0.48 |  | 22.38 (6.49) | 28.00 (2.83) | .22 | 1.12 |
|  | **Depression** | | | |  | **Depression** | | | |
|  | **No** | **Yes** |  |  |  | **No** | **Yes** |  |  |
|  | **n=2105** | **n=28** |  |  |  | **n=673** | **n=15** |  |  |
|  | **Mean (SD)** | **Mean (SD)** | ***P*-value** | **Cohen d effect Size** |  | **Mean (SD)** | **Mean (SD)** | ***P*-value** | **Cohen d effect Size** |
| **Fear Scale** | 23.94 (6.63) | 27.29 (7.16) | .01 | 0.49 |  | 22.44 (6.50) | 20.67 (5.90) | .30 | 0.29 |
|  | **Anxiety** | | | |  | **Anxiety** | | | |
|  | **No** | **Yes** |  |  |  | **No** | **Yes** |  |  |
|  | **n=2104** | **n=29** |  |  |  | **n=673** | **n=15** |  |  |
|  | **Mean (SD)** | **Mean (SD)** | ***P*-value** | **Cohen d effect Size** |  | **Mean (SD)** | **Mean (SD)** | ***P*-value** | **Cohen d effect Size** |
| **Fear Scale** | 23.92 (6.62) | 28.69 (6.45) | .01 | 0.73 |  | 22.46 (6.46) | 19.67 (7.51) | .10 | 0.40 |
|  | **Insomnia** | | | |  | **Insomnia** | | | |
|  | **No** | **Yes** |  |  |  | **No** | **Yes** |  |  |
|  | **n=2066** | **n=67** |  |  |  | **n=665** | **n=23** |  |  |
|  | **Mean (SD)** | **Mean (SD)** | ***P*-value** | **Cohen d effect Size** |  | **Mean (SD)** | **Mean (SD)** | ***P*-value** | **Cohen d effect Size** |
| **Fear Scale** | 23.90 (6.65) | 26.73 (5.95) | .01 | 0.45 |  | 22.39 (6.47) | 22.70 (7.13) | .82 | 0.05 |

| **Supplementary Table 4: Known-group comparison by multiple linear regression (N=2821)** | | |
| --- | --- | --- |
|  | **β (95% confidence interval)** | ***P*-value** |
| Hong Kong (vs. Living in mainland China) | 1.36 (0.78,1.95) | <.001 |
| 60 years or older (vs. 18-59 years) | 1.66 (0.75,2.58) | <.001 |
| Male (vs. female) | -0.44 (-0.93,0.06) | 0.08 |
| Hypertension | 1.58 (0.72,2.45) | <.001 |
| Diabetes | -0.64 (-1.77,0.50) | 0.27 |
| Liver diseases | 2.18 (0.32,4.03) | 0.02 |
| Heart diseases | 1.51 (-0.19,3.21) | 0.08 |
| Stroke | 0.74 (-2.32,3.81) | 0.63 |
| Chronic obstructive pulmonary disease | 1.36 (-0.79,3.52) | 0.21 |
| Cancer | 2.37 (-0.89,5.62) | 0.15 |
| Depression | 0.70 (-1.35,2.74) | 0.50 |
| Anxiety | 1.51 (-0.54,3.57) | 0.15 |
| Insomnia | 1.74 (0.31,3.16) | 0.02 |
| R^2^ | 0.04 |  |
